# Computational Insights of Unfolding of N-Terminal Domain of TDP-43 Reveal the Conformational Heterogeneity in the Unfolding Pathway

**DOI:** 10.3389/fnmol.2022.822863

**Published:** 2022-04-25

**Authors:** Ruiting Li, Ruhar Singh, Tara Kashav, ChunMin Yang, Ravi Datta Sharma, Andrew M. Lynn, Rajendra Prasad, Amresh Prakash, Vijay Kumar

**Affiliations:** ^1^School of Engineering, Guangzhou College of Technology and Business, Guangzhou, China; ^2^School of Computational & Integrative Sciences, Jawaharlal Nehru University, New Delhi, India; ^3^Department of Life Science, Central University of South Bihar, Gaya, India; ^4^Amity Institute of Biotechnology, Amity University Haryana, Gurgaon, India; ^5^Amity Institute of Integrative Sciences and Health (AIISH), Amity University Haryana, Gurgaon, India; ^6^Amity Institute of Neuropsychology & Neurosciences (AINN), Amity University, Noida, India

**Keywords:** TDP-43, NTD, unfolding intermediates, DMSO, conformational heterogeneity

## Abstract

TDP-43 proteinopathies is a disease hallmark that characterizes amyotrophic lateral sclerosis (ALS) and frontotemporal lobar degeneration (FTLD). The N-terminal domain of TDP-43 (NTD) is important to both TDP-43 physiology and TDP-43 proteinopathy. However, its folding and dimerization process is still poorly characterized. In the present study, we have investigated the folding/unfolding of NTD employing all-atom molecular dynamics (MD) simulations in 8 M dimethylsulfoxide (DMSO) at high temperatures. The MD results showed that the unfolding of the NTD at high temperature evolves through the formation of a number of conformational states differing in their stability and free energy. The presence of structurally heterogeneous population of intermediate ensembles was further characterized by the different extents of solvent exposure of Trp80 during unfolding. We suggest that these non-natives unfolded intermediate ensembles may facilitate NTD oligomerization and subsequently TDP-43 oligomerization, which might lead to the formation of irreversible pathological aggregates, characteristics of disease pathogenesis.

## Introduction

The mislocalization and cytoplasmic ubiquitinated inclusions of transactivation response DNA-binding protein 43 kDa (TDP-43) are the major neuropathological hallmark in Amyotrophic lateral sclerosis (ALS) and frontotemporal dementia (FTD; Neumann et al., 2006; Mackenzie et al., 2010). TDP-43 is also associated with tau-negative frontotemporal lobar degeneration (Liscic et al., 2008), and in the cases with Alzheimer’s (AD), Parkinson’s (PD), and Huntington’s (HD) diseases (Rohn, 2008; Schwab et al., 2008; Chanson et al., 2010), although they are not considered as a primary target in these cases. TDP-43 consists of an N-terminal domain (NTD: residues 1–76) with a well-defined globular folded structure, two highly conserved RNA recognition motifs (RRM1: 106–176 and RRM2: 191–259), and an unstructured, glycine-rich C-terminal domain (CTD: 274–414) which harbor most of the pathologically linked ALS-mutations (Buratti, 2015) and display greater ability to self-assemble, phase separate, and aggregate (Colombrita et al., 2009). TDP-43 is involved in many different physiological functions including transcription, translation, splicing (Ayala et al., 2005; Casafont et al., 2009; Lagier-Tourenne et al., 2010), microRNA processing (Gregory et al., 2004), apoptosis (Sreedharan et al., 2008), cell division (Ayala et al., 2008), axonal transport (Casafont et al., 2009) and embryo development (Sephton et al., 2010).

The NTD plays a crucial role in the structure and oligomerization of the full-length TDP-43 protein. NTD has the propensity to dimerize that can assemble into reversible higher-order oligomers, involved in splicing activity and in liquid-liquid phase separation. The NTD-driven oligomerization modulates the physiological function of TDP-43 and also participates in driving TDP-43 aggregation into pathological inclusions, revealing a dichotomy in TDP-43 (Zhang et al., 2013; Afroz et al., 2017; Jiang et al., 2017; Tsoi et al., 2017; Wang et al., 2018). Thus, the stable and well folded NTD mediates the formation of physiological oligomers whereas, destabilized and unfolded NTD results in the formation of pathological inclusions (Qin et al., 2014; Chang and Huang, 2016).

Tsoi et al. (2017) reported the unfolding of NTD at low pH proceeds with the presence of several intermediate states. We have also previously shown the presence of different stable and meta-stable intermediate states during the unfolding of NTD in 8 M urea at 300 K–500 K (Prakash et al., 2018a). Through all-atom MD simulation, we have studied the stability and dynamics of the NTD mutations, L27A, L28A, and V31R, and provide insights into the mechanisms of the destabilization of NTD variants (Kumar et al., 2019). A recent study also demonstrated the presence of different partially folded states during the folding pathways of NTD (Vivoli-Vega et al., 2020). Therefore, the folding dynamics and confirmational stability studies of NTD are essential to underpinning the role of folding intermediates behind the dichotomy of TDP-43.

In this work, we studied the unfolding process of NTD in 8 M DMSO at different temperatures, i.e., 300 K, 350 K, 400 K, 450 K, and 500 K. The results of the cumulative microsecond simulations indicate that the unfolding pathway of NTD is characterized by the presence of different partially folded stable intermediates which may play an important role in mediating TDP-43 self-assembly into pathological oligomers.

## Methods

Using GROMACS v5.1.4 (Wennberg et al., 2015), MD simulations were conducted for N-terminal TDP-43 (PDB ID: 2N4P) in aqueous DMSO at different temperatures: 300 K, 350 K, 400 K, 450 K, and 500 K. The force field is selected as CHARMM27 (Sapay and Tieleman, 2011) and the cubic simulation boxes were filled TIP3P water molecules, having the padding size of 10 Å to fully submerged the protein. For the electrically neutralized system, physiological ion concentrations of 0.15 M counter-ions (Na^+^ and Cl^−^) were added to each simulation box (Kumar et al., 2018; Prakash et al., 2019) and periodic boundary conditions were defined for x, y, and z directions. CHARMM-GUI server was used to parametrize the co-solvent DMSO 8 M as defined in the previously published approaches (Banerjee et al., 2010; Kim et al., 2017; Singh et al., 2020). The prepared simulation boxes (59 Å^3^), having DMSO molecules 1,040 were allowed for energy minimized up to 50,000 steps applying steepest descent and conjugant gradients, respectively. Two steps ensemble process, NVT, and NPT were performed up to 1 nm to equilibrate the prepared systems. Berendsen thermostat (Berendsen et al., 1987) and Parrinello-Rahman pressure (Parrinello and Rahman, 1980) were applied to maintain the temperature and pressure. LJ potential applied for van der Waals interactions (Cisneros et al., 2014) and the long-range electrostatic interactions were taken care of by particle mesh Ewald (PME). The ensemble structure obtained from NPT was subjected to a production run of 500 ns on DELL T640 machine enabled GPU V100 (Prakash et al., 2021) with an integration time step 2. The atomic energies, velocities, and trajectories were updated at the time interval of 10 ps.

### MD Trajectory Analysis

Different conformational order variables such as root mean square deviation (RMSD), radius of gyration (*Rg*), solvent accessible surface area (SASA), and root mean square fluctuation (RMSF) were evaluated by Gromacs utilities with MDTraj (Mcgibbon et al., 2015). MDTraj and python script were applied for native contact (Nc) and free energy landscape (FEL) calculation as described in our previous studies (Kumar et al., 2019; Prakash et al., 2019, 2021).

### Fraction of Native Contacts

The structure of a protein is largely stabilized by the native contacts which play a significant role in determining the folding mechanism, thus, the loss of native contacts is indicative of the protein unfolding process. For the protein folding study the fraction of native contacts, Nc, define as the contact distance between any two heavy pair atoms from two different residues, r_i_, and r_j_, as |r_i_ − r_j_| ≤ 4 Å (Prakash et al., 2018a; Kumar et al., 2019).

### Free Energy Landscape

This statistical analysis of protein folding pathways was performed through the free energy landscape (FEL). FEL was studied by applying the equation for Boltzmann inversion, *F* = −RT ln P, where, P define as the joint probability of structural order parameters (Prakash et al., 2018a; Kumar et al., 2019). In this study, we described FEL as a function of two different structural order variables, including RMSD and fraction of native contacts (Nc). To further validate the results, we also study FEL described as a function of *Rg* and Nc.

## Results

To monitor the thermal unfolding of NTD, several unconstrained all-atom MD simulations of NTD in the presence of aqueous 8 M DMSO solution at different temperatures (300 K, 350 K, 400 K, 450 K, and 500 K) for 500 ns each were performed. The different structural order parameters were then analyzed to allow a detailed description of the NTD unfolding. We have previously reported the structural stability of NTD in water and in 8 M urea at 300 K (Prakash et al., 2018a). At 300 K, the Cα-RMSD of NTD did not change largely and remained stabilized around a value of <1.0 nm. The other structural parameters like *Rg*, RMSF, and SASA did not show significant changes and remained stable also. However, a slight decrease in Nc of NTD was observed during the simulation. Thus, to accelerate the unfolding process of NTD, the all-atom MD simulations in the presence of 8 M DMSO for 500 ns were carried out with higher temperatures, very similar to our previous studies (Prakash et al., 2018a, b).

### Temperature-Induced Changes of Conformational Dynamics

As a first step in elucidating the unfolding events of NTD, we here studied the thermal dependence of different structural order parameters obtained from MD trajectories in the temperature range of 300 K to 500 K.

**Figure 1:**
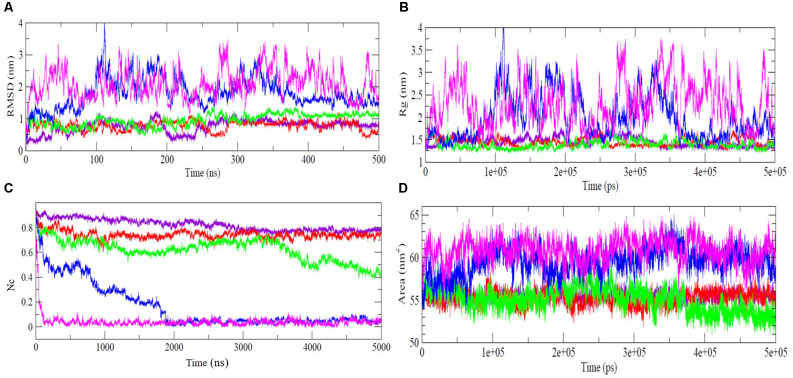
Thermal unfolding analysis of NTD. Time evolutions of **(A)** Cα- RMSD, **(B)** radius of gyration, *Rg*, **(C)** fraction of native contacts, Nc, and **(D)** SASA of NTD at temperature 300 K (indigo), 350 K (red), 400 K (green), 450 K (blue), and 500 K (purple). NTD, N-terminal domain; SASA, solvent accessible surface area.

In the MD trajectory run at 300 K, 350 K, and 400 K, the RMSD did not show any significant increase indicating structural stability up to 400 K (Figure 1A). However, a slight increase in RMSD has been observed in the last ~125 ns of simulation, implying marginal structural changes at this temperature. At 450 K, the RMSD values increase gradually during the 100 ns simulations and exhibit a maximum deviation of ~4.0 nm around 100 ns and then decrease back to ~2.0 nm around 140 ns and further underwent significant fluctuations till ~400 ns. The RMSD reaches a value of ~1.5 nm and remains stable during the last 100 ns of simulation, indicating that the NTD structure undergoes constant evolution at high temperatures. However, NTD underwent robust structural changes at 500 K, indicating the complete unfolding of the protein. At higher temperatures, continuous fluctuations of RMSD indicate that NTD adopts expanded flexible conformations during the simulation. The average RMSD values at different temperatures are reported in Table 1 which also indicates a significant increase in RMSD values at higher temperatures. Thus, the results clearly suggest that NTD underwent significant structural transformations at higher temperatures with an overall higher RMSD. This is in agreement with the observed unfolding of NTD in the presence of 8 M urea (Prakash et al., 2018a).

**Table 1:** Structural order parameters (average ± standard deviation) obtained from MD simulations of NTD unfolding in the presence of 8 M DMSO at different temperatures.

Temperature (K)	RMSD (nm)	*Rg* (nm)	Nc	SASA (nm^2^)
300	0.76 ± 0.19	1.44 ± 0.09	0.82 ± 0.04	55.47 ± 0.63
350	0.78 ± 0.15	1.46 ± 0.08	0.74 ± 0.03	55.20 ± 0.85
400	0.98 ± 0.19	1.49 ± 0.10	0.52 ± 0.12	55.02 ± 1.43
450	1.79 ± 0.47	1.98 ± 0.49	0.10 ± 0.13	59.50 ± 1.60
500	2.09 ± 0.45	2.30 ± 0.51	0.04 ± 0.05	60.86 ± 1.41

RMSD, root mean square deviation; NTD, N-terminal domain; DMSO, dimethylsulfoxide; SASA, solvent accessible surface area.

*Rg* provides important insights into the protein structural compactness. Similar to the RMSD trajectory, the Rg trajectory shows that NTD maintains relatively stable *Rg* at 300 K, 350 K, and 400 K simulations (Figure 1B). At 450 K, the *Rg* value starts increasing from ~80 ns and reaches a maximum value of 4.0 nm at 110 ns. The steady increment in *Rg* at this temperature indicates the structural expansion of the protein and suggests that NTD explored several conformational states during unfolding. From 100 ns to 350 ns, the *Rg* trajectory showed fluctuations that attained stability towards the last 100 ns of simulation. At 500 K, a larger fluctuation in *Rg* is evident which indicates the expansion of the protein molecules with disruption of secondary and tertiary structures (Figure 1B). Importantly, it should be noted that NTD attains expanded flexible conformations at 450 K and 500 K which is evident from the large fluctuations of *Rg* values. As quantified, the average *Rg* values of NTD are around 1.44 nm, 1.46 nm, 1.49 nm, 1.98 nm, and 2.30 nm, at 300 K, 350 K, 400 K, 450 K, and 500 K, respectively (Table 1).

Thus, the *Rg* trajectory suggests that NTD unfolds at higher temperatures and adopts an expanded flexible conformation due to the significant loss of a large fraction of native tertiary contacts. Loss of native contacts is accompanied with protein unfolding and can also provide evidence of the presence of intermediate states in the unfolding pathway. The time evolutions of the Nc retained by NTD at different temperatures are given in Figure 1C. It is clear that about 80% of native contacts are retained at 300 K, and 350 K. With the further increase in the temperature, the protein starts losing a large fraction of the native contacts indicate the unfolding of the protein. At 450 K, complete loss of tertiary contacts occurs after ~200 ns of simulation while, at 500 K, about 10%–15% of the native contacts were retained only after 10 ns indicating the complete unfolding of NTD, as was also shown from RMSD and *Rg* results (Figures 1A,B).

In addition, the SASA for NTD was calculated to monitor the structural changes of NTD at different temperatures (Figure 1D, Table 1). As can be seen from Figure 1D and Table 1, the SASA value at 300 K is 55.47 nm^2^ and remained unchanged till 400 K. With the further increase of temperature, the SASA increased up to max average values of 59.50 nm^2^, and 60.86 nm^2^ at 450 K and 500 K, respectively. In another word, the unfolding transitions of NTD occurred much faster at high-temperature simulations.

To gain further insight into the residual flexibility changes with increasing temperature, we calculated the RMSF along the amino acid sequence (Supplementary Figure 1). We did not observe much significant change in flexibility of the protein at 300 K and 350 K. However, at 400 K, except the very flexible N-terminal His-tag, the C-terminal region of the protein (residues ~65–80) is highly flexible. The β-hairpin forming residues 55–62 is comparatively rigid. The maximum increase in fluctuations is observed in the β4-turn-β5 region. The increase in RMSF of C-terminal turn regions has also been shown in previous studies (Prakash et al., 2018a; Kumar et al., 2019).

**Figure 2:**
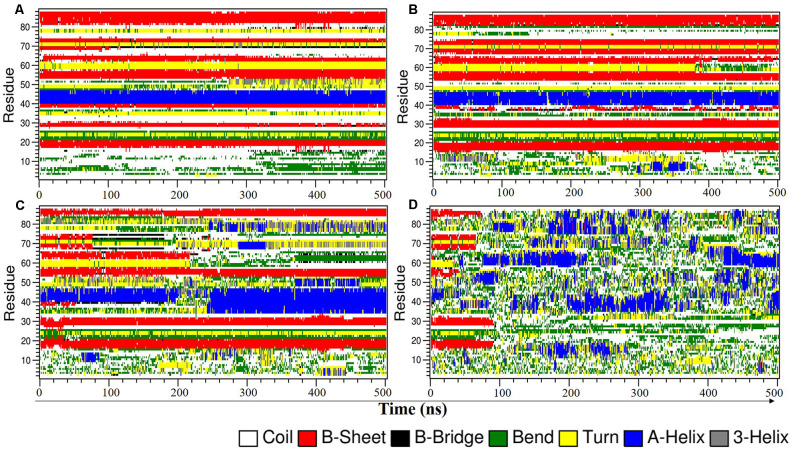
Time evolution plot of NTD secondary structure using DSSP in 8 M DMSO at **(A)** 300 K, **(B)** 350 K, **(C)** 400 K, and **(D)** 450 K. DMSO, dimethylsulfoxide.

To further understand the effect of 8 M DMSO at different temperatures to the unfolding of NTD in more detail, the time evolution of the secondary structure profiles of NTD are depicted in Figure 2. The NTD (PDB id:2N4P) adopts a compact fold consist of six β-strands, one α-helix and two β-hairpins. At 300 K, the overall secondary structures are maintained throughout the simulation (Figure 2A). Even at 350 K, only minor structure changes were observed with the appearance of additional helix and turn/bend structure at N-terminal during the simulation (Figure 2B). Significant changes in the secondary structure profile of NTD were observed at higher temperature (Figures 2C,D). At 400 K, the significant changes observed are the loss of helix along with loss of C-terminal β-strands (Figure 2C). We also observed the appearance of helices during the simulation both at 400 K and 450 K. At 450 K, the secondary structure lost completely along with appearance of helices, turn and bend throughout the simulation (Figure 2D). Overall, decrease in helical content and increase in irregular secondary structures content like bend and turn along with loss of β strands characterized the unfolded conformation of NTD at higher temperature. Furthermore, snapshots of the NTD structure (Supplementary Figures 2, 3) obtained at higher temperature (i.e., 400 K) demonstrated the significant loss of β strands along with appearance of additional α-helix (Supplementary Figure 3).

### Free-Energy Landscape of NTD Reveals the Presence of Stable Intermediates

Free energy landscape (FEL) has been used for describing conformational changes associated with protein unfolding/folding pathways. Even though the protein’s free energy landscape can be rugged and high dimensional (Frauenfelder et al., 1991), it can frequently indicate the predominant lesser energy regions and can reveal pathways for conformational changes during protein unfolding.

**Figure 3:**
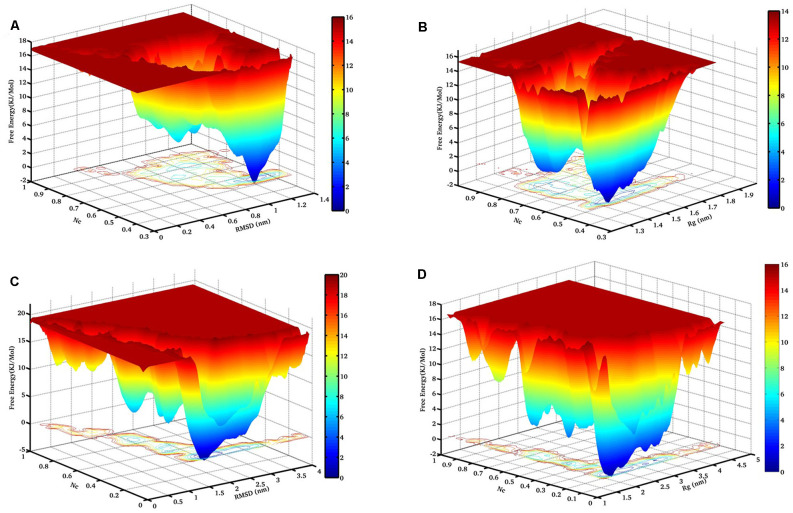
The free energy landscape (FEL) of NTD during thermal unfolding. Free energy contour maps constructed at 400 K from **(A)** Nc vs. Cα-RMSD, and **(B)** Nc vs. *Rg*. Similarly, FEL at 450 K from **(C)** Nc vs. Cα-RMSD, and **(D)** Nc vs. *Rg*. The color is scaled according to kcal mol^−1^. The dark blue region represents the energy minima and energetically favored protein conformations, and the yellow region represents the unfavorable high-energy conformations. RMSD, root mean square deviation.

To map the unfolding pathway in 8 M DMSO at 400 K and 450 K, FEL contour maps were constructed from the joint probability distribution (P) of Nc with RMSD and *Rg* using the equation, *F* = −RT ln P. The FEL plot clearly indicated the transition from the native folded state to the unfolded state through a minimum free energy pathway consisting of different stable intermediates (Figure 3). The FEL plot at higher temperature showed the unfolding transition from the native folded state to the unfolded state through a minimum energy pathway characterized by the presence of a number of unfolding intermediates (Figure 3). At 400 K, the highly rugged FEL showed the existence of two main free energy basins in the global free energy minimum region of the FEL, indicating the presence of two main conformational metastable states (Figures 3A,B). One of the intermediate states has higher free energy and is characterized by the presence of deeper and wider basins indicating that it has a relatively larger population and is more stable. The FEL at 450 K displayed one wide global minima in addition to two well-resolved local minima (Figures 3C,D). The FEL plot also has several high-energy destabilized intermediates, overall indicating the presence of several intermediates in the unfolding pathway of NTD.

Moreover, we have examined two FEL contour maps where the first FEL was calculated as a function of the Nc and RMSD pair and the second FEL was calculated as a function of Nc and *Rg*. Interestingly, the significant overlap between these two FELs reveals excellent agreement for the reported configurations.

**Figure 4:**
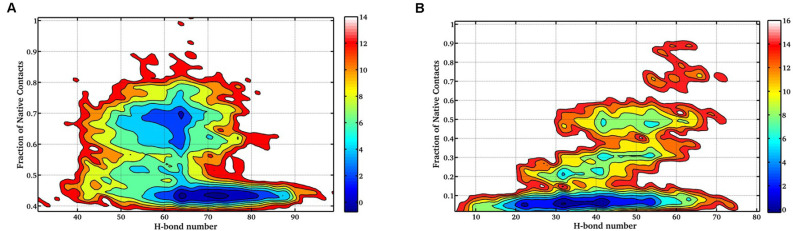
FEL of the number of intraprotein H- bonds vs. the number of native contacts at **(A)** 400 K, and **(B)** 450 K. The results clearly indicate the large decrease in the number of H-bonds at a higher temperature.

Next, the FEL involving the number of intraprotein hydrogen (H)-bonds and Nc at 400 K and 450 K was constructed as shown in Figure 4. At 400 K, the number of intraprotein H-bonds decreased from ~80% to ~60% along the NTD unfolding pathway whereas, the number of native contacts decreased from ~70% to ~40% (Figure 4A). However, at 450 K, there is no significant decrease in the number of intraprotein H-bonds, but the number of native contacts reduced to ~10% along the unfolding pathway (Figure 4B). These results thus convincingly elucidate the role of non-native interactions, mostly H-bonds, in stabilizing the unfolding intermediate states.

### SASA vs. RMSD Analysis

**Figure 5:**
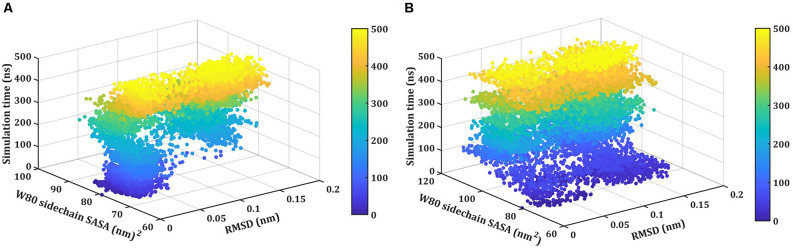
Thermal unfolding of NTD in 8 M DMSO. Time evolution of the solvent-exposed surface area, SASA, of the W80 side chain and Ca-RMSD at **(A)** 400 K, and **(B)** 450 K. Each point on this plot is colored according to its time of occurrence according to the color scale shown.

Next, we monitor the unfolding pathway of NTD at 400 K through the analysis of the evolution of the side chain SASA of W80 along with the Ca RMSD during 500 ns simulation (Figure 5A). In the native state, W80 is solvent-exposed, with a side chain SASA of ~70 nm^2^. At 400 K, W80 undergoes different extents of solvent exposure during unfolding, similar to the experimentally observed decrease in fluorescence (Mompean et al., 2016; Tsoi et al., 2017). During the first 150 ns, the SASA value ranges from ~70 to 85 nm^2^ indicating the presence of a native-like ensemble with RMSD ≤0.08 nm. The side chain SASA of W80 increased as NTD unfolds further during the last 300 ns of simulation. This unfolded intermediate ensemble is populated with an RMSD of ~0.20 nm with the SASA of ~100 nm^2^. This unfolded state displays the transition of W80 between partially buried and solvent-exposed states. At 450 K, the change in SASA of W80 is evident from starting of the simulation (Figure 5B). During the first ~100 ns of simulation, W80 undergoes SASA of ~100 nm^2^ with RMSD of ~0.1 nm. The SASA value increases further (~120 nm^2^) along with the increase in RMSD (~0.2 nm) during the simulation, suggesting the complete unfolding of the protein. These results, therefore, show that solvent exposure of W80 can occur on different time scales and is also depends on the temperature, indicating that the unfolding reaction is non-cooperative and is not all-or-none. However, it is not yet clear that the observed non-cooperativity is either due to the presence of a high concentration of DMSO or increased temperature.

### DCCM Analysis

**Figure 6:**
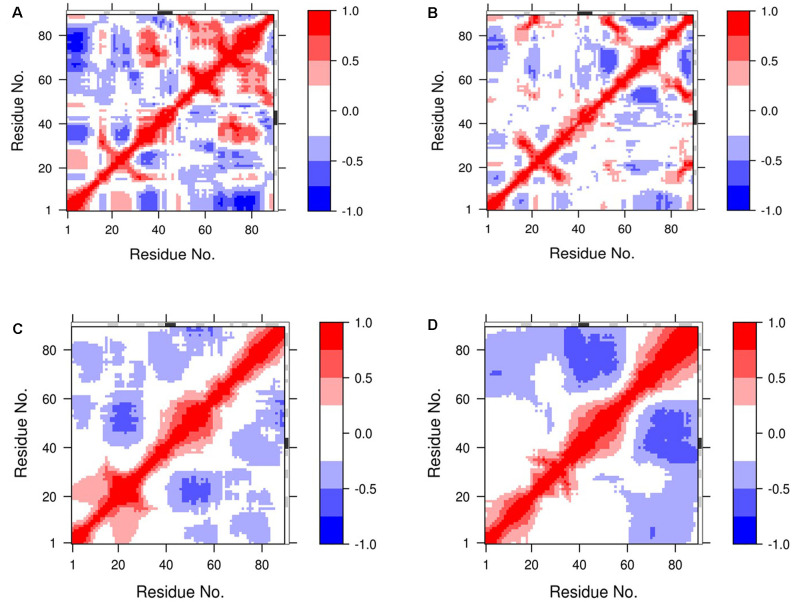
Dynamic cross-correlation map (DCCM) of NTD’s residue motion during MD simulation. The residue motions of NTD at **(A,B)** 400 K, and **(C,D)** 450 K during the start and the end of the simulation, respectively. The degrees of the correlation motions and anti-correlation motions are shown in blue and red, respectively.

The dynamic cross-correlation matrices (DCCM) analysis was further studied to illustrate dynamical information of proteins in two dimensions (Pandey et al., 2020; Ahamad et al., 2022; Figure 6). The positive red regions in the correlation maps signify a strongly correlated motion of the residues in the same direction whereas, the negative blue regions indicate strong anti-correlated motion of the residues. As shown in Figure 6, both the correlated and anti-correlated motions of the residues disrupted in 8 M DMSO with the temperature. From the comparison of the correlation map, a substantial loss of motions was observed at a higher temperature. The loss of correlation in the motion thus hinted toward the loss of contact among the residues, which lead to the destabilization and unfolding of the protein.

## Discussion and Conclusion

The prion-like CTD is largely involved in the aggregation of TDP-43 and most of the fALS mutations are located within this domain (Li et al., 2018; Prasad et al., 2019). However, many reports also suggest that NTD plays a crucial role in regulating the assembly of TDP-43. Biophysical techniques indicated that TDP-43 converts into amyloid-like aggregates either through CTD (Tsoi et al., 2017) or through NTD (Afroz et al., 2017; Tsoi et al., 2017). Afroz et al. (2017) also suggested that the NTD-driven homo-oligomerization might exert a protective role against pathological aggregation. Be it physiological or pathological, the NTD-mediated oligomerization seems to play a major role in the aggregation properties of TDP-43 and, therefore, the identification of possible conformational ensembles populated during the unfolding pathway of NTD can help us to understand the behavior of the TDP-43.

We have previously investigated the thermal unfolding of NTD in the presence of 8 M urea using all-atom MD simulations and showed that unfolding of NTD at 350 K occurs through the presence of different stable and meta-stable intermediate states. These unfolded intermediates share the properties of molten globule states stabilized largely by non-native hydrophilic interactions and are highly energetically frustrated. The non-native unfolded intermediate states might facilitate protein oligomerization and aggregation. Very recently, Vivoli-Vega et al. (2020) biophysically characterized the folding/dimerization of NTD and identified a head-to-tail arrangement during the dimerization of NTD. The authors also found that the folding of NTD proceeds through the formation of a collapsed state and an intermediate state. Thus, these studies showed that NTD is a highly plastic protein and prone to populate different conformational ensembles and oligomeric states which may enable NTD to control the assembly state of TDP-43. The identification of conformational ensembles populated transiently or permanently during the protein folding/unfolding events is critical to understanding the factors affecting the equilibrium between folding and misfolding pathways.

Different chemical denaturing agents act differently on the protein unfolding process (Mukherjee et al., 2019; Singh et al., 2020). The purpose of the unfolding study here to determine the structural stability of different intermediate stages occurs through the unfolding pathway of NTD which is a β-protein, consist of six β-sheets and one small α-helix. Several studies suggest that protein unfolding in urea happened through the direct interaction with polar amino acids, which are present at the surface. The formation of hydrogen bonds lead to the disruption of the native intramolecular hydrogen bonding network and hydrophobic residues subsequently exposure to solvent (Rocco et al., 2008; Roy and Bagchi, 2013; Dasgupta et al., 2014; Mukherjee et al., 2019). Other approaches indicate that the urea induced stress is the major driving forces of protein unfolding which result in the dispersion of water molecules around the protein and core structure of protein is disrupted as the hydrophobic interactions are solvent exposed (Day et al., 2002; Dyson et al., 2006; Rocco et al., 2008; Roy and Bagchi, 2013). Whereas DMSO as chemical agent may play the various roles as stabilizer, inhibitor, activator, and cryoprotector (Pegg, 2007; Awan et al., 2020; Gironi et al., 2020). Furthermore, the secondary structure of β-sheets melted preferentially in urea denaturant. Oppositely, DMSO may act as secondary structure β-sheet stabilizer (Roy and Bagchi, 2013; Gironi et al., 2020). However, the challenging task with the protein folding/unfolding study is to track the consequence of conformational ensembles which occurs through the unfolding pathway. The adding of temperature to MD simulation accelerates the unfolding process rather have no change on the protein folding/unfolding pathway (Day et al., 2002; Daggett and Fersht, 2003).

With this view, we here studied the unfolding of NTD as the structural and conformational integrity of the NTD is essential for the proper function of TDP-43. A detailed account of the unfolding and thermodynamics of the NTD has been delineated in 8 M DMSO at high temperatures using all-atom MD simulations. The unfolding process of NTD is characterized by the presence of different meta-stable intermediates at 400 K and 450 K along with the population of completely unfolded species at 450 K. The structural transitions during the unfolding indicated the appearance of helices along with increase in minor secondary structures like bend or turn. Thus, the computational insights of the unfolding pathway of NTD will help in understanding the full description of the TDP-43 folding/unfolding landscape.

## Data Availability Statement

The raw data supporting the conclusions of this article will be made available by the authors, without undue reservation.

## Author Contributions

VK and AP designed the study. RL RS, and AP performed the experiments and calculations. RL, TK, AP, and VK analyzed the data. CY, RDS, AP, and VK wrote the main text with the contributions of AL and RP. All authors contributed to the article and approved the submitted version.

## Conflict of Interest

The authors declare that the research was conducted in the absence of any commercial or financial relationships that could be construed as a potential conflict of interest.

## Publisher’s Note

All claims expressed in this article are solely those of the authors and do not necessarily represent those of their affiliated organizations, or those of the publisher, the editors and the reviewers. Any product that may be evaluated in this article, or claim that may be made by its manufacturer, is not guaranteed or endorsed by the publisher.

## Supplementary Material

Supplementary Figure 1 Positional root-mean-square fluctuations (RMSF) of the backbone Cα-atoms for NTD at diffrent temperature. RMSF at 300 K (indigo), 350 K (red), 400 K (green), 450 K (blue), and 500 K (purple).

Supplementary Figure 2 Structural snapshots of different conformational states observed during the unfolding pathway of the NTD in 8 M DMSO at 350 K.

Supplementary Figure 3 Structural snapshots of different conformational states observed during the unfolding pathway of the NTD in 8 M DMSO at 400 K.
