# Supplementary material for: Computational Insights of Unfolding of N-Terminal Domain of TDP-43 Reveal the Conformational Heterogeneity in the Unfolding Pathway
Source: Front Mol Neurosci. 2022 Apr 25;15:822863. doi: 10.3389/fnmol.2022.822863 (PMC9083116; doi:10.3389/fnmol.2022.822863)
Supplement: Supplementary Figure 1 — Positional root-mean-square fluctuations (RMSF) of the backbone Cα-atoms for NTD at diffrent temperature. RMSF at 300 K (indigo), 350 K (red), 400 K (green), 450 K (blue), and 500 K (purple). [file Data_Sheet_1.PDF]

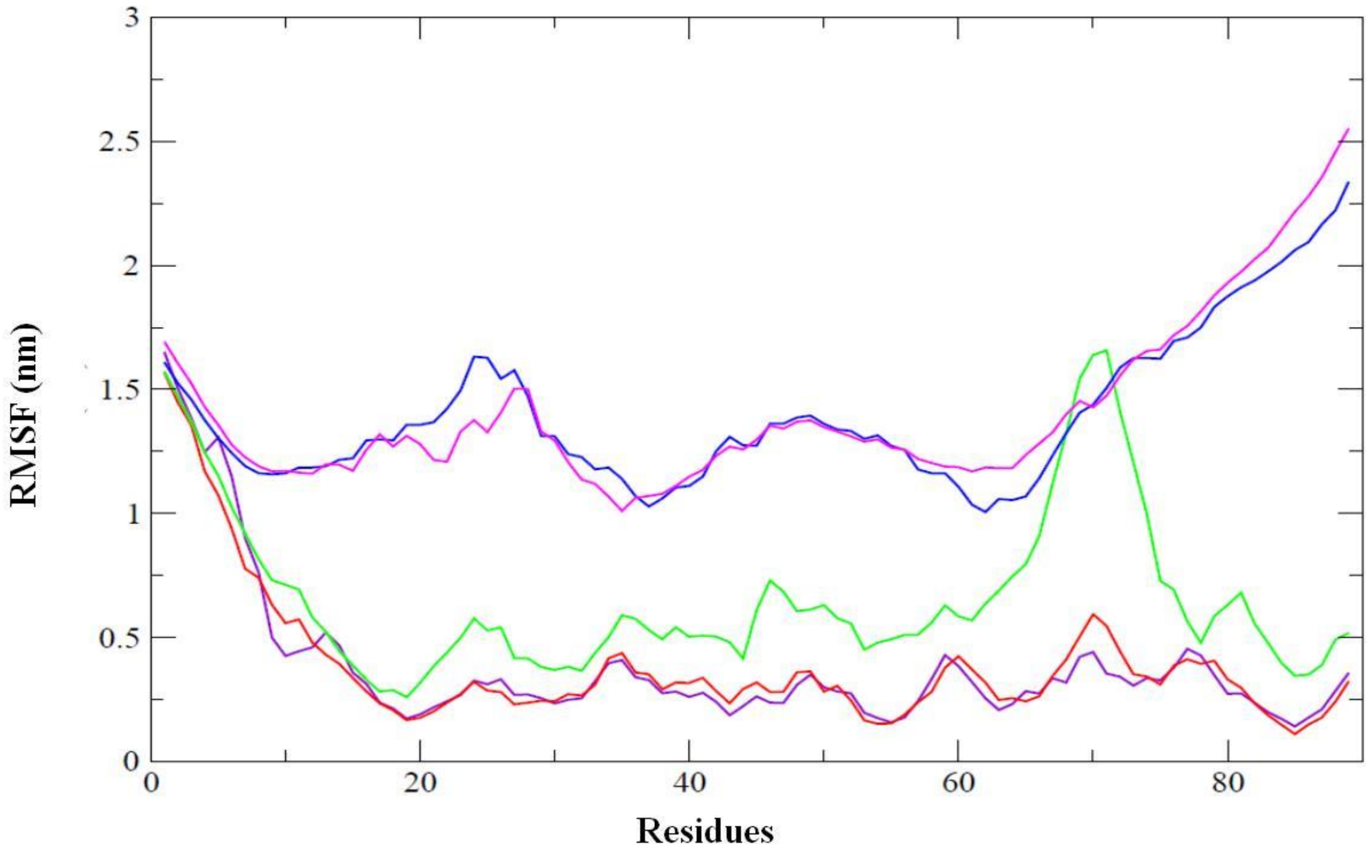

Supporting Information Figure 1. Positional root-mean-square fluctuations (RMSF) of the backbone Cα-atoms for NTD at different temperature. RMSF at 300 K (indigo), 350 K (red), 400 K (green), 450 K (blue), and 500 K (purple).

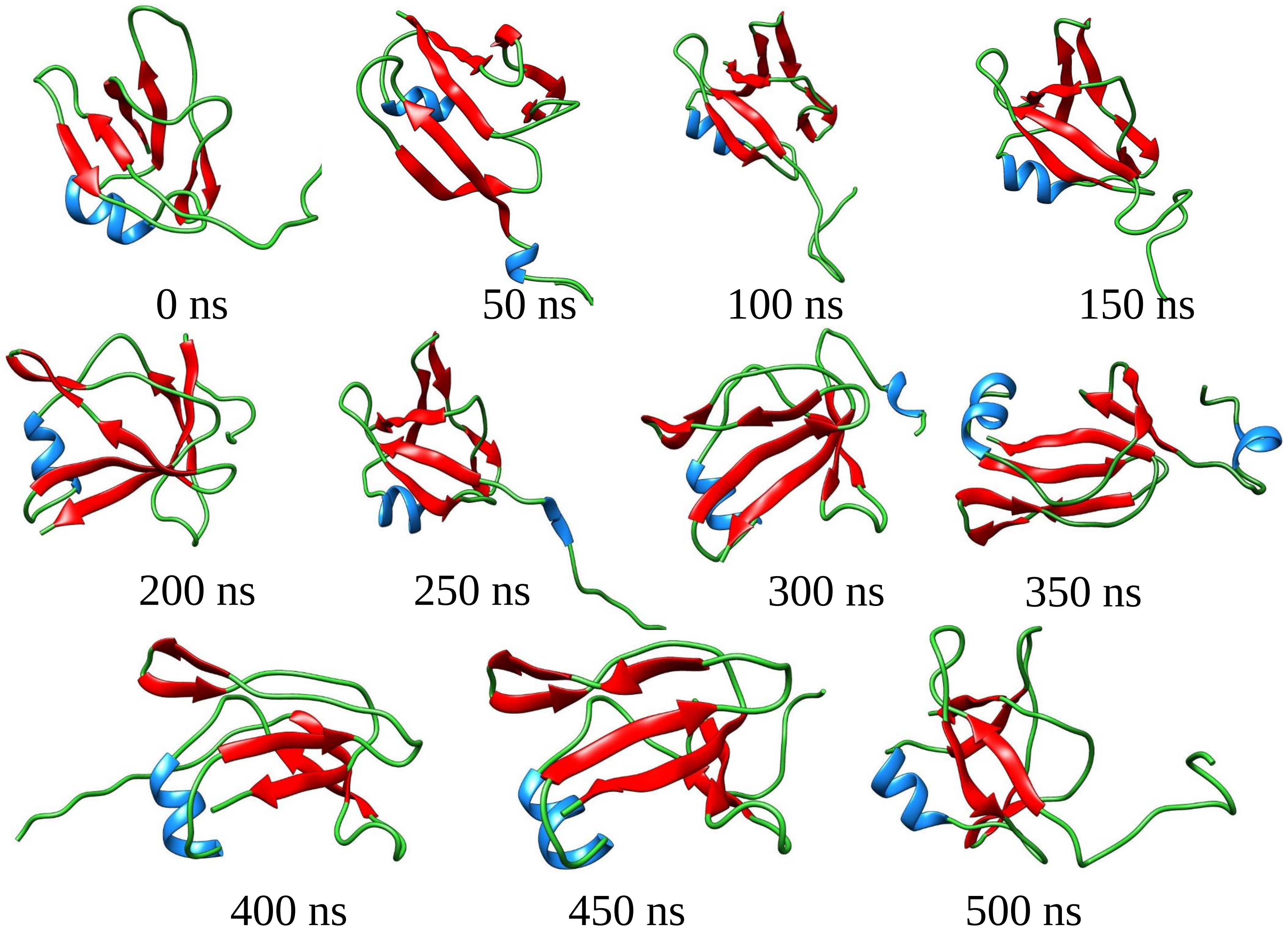

Supporting Information Figure 2. Structural snapshots of different conformational states observed during the unfolding pathway of the NTD in 8M DMSO at 350 K.

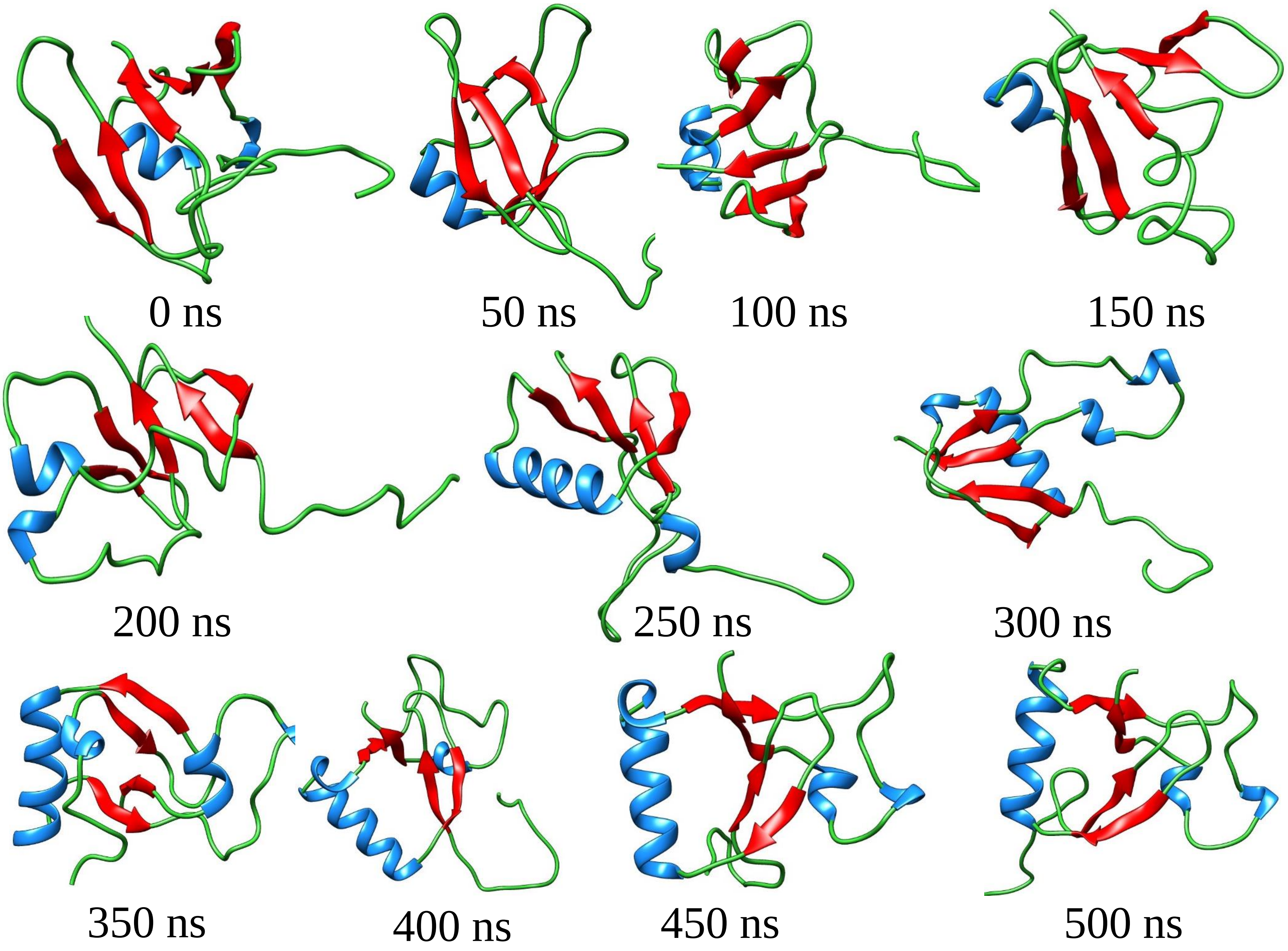

Supporting Information Figure 3. Structural snapshots of different conformational states observed during the unfolding pathway of the NTD in 8M DMSO at 400 K.
